# Estimating Impact of SARS-CoV-2 Infection on Health-Related Quality of Life Among Persons Aged 8 Years and Older, August 2020–July 2022

**DOI:** 10.36469/001c.163131

**Published:** 2026-07-28

**Authors:** Sheroi Johnson, Huong Q. Nguyen, Melissa S. Stockwell, Christina A. Porucznik, Ruth A. Karron, Fatimah S. Dawood, Lisa A. Posser, Alisha P. Sarakki, Joseph B. Stanford, Marissa Hetrich, Elizabeth Schappell, Maria D. Knoll, Vic Veguilla, Alexandra Mellis, Jazmin Duque, Zuha Jeddy, Jamison Pike, Melissa A. Rolfes

**Affiliations:** 1 Centers for Disease Control and Prevention, Atlanta, Georgia; 2 Marshfield Clinic Research Institute, Marshfield, Wisconsin; 3 Columbia University, New York, New York; 4 University of Utah, Salt Lake City, Utah; 5 Johns Hopkins University, Baltimore, Maryland; 6 University of Michigan, Ann Arbor, Michigan; 7 Abt Associates, Rockville, Maryland

**Keywords:** SARS-CoV-2, health quality of life, vaccination, EQ-5D

## Abstract

**Background:** Severe COVID-19 results in substantial economic burden and impacts quality of life. Assessing how mild COVID-19 impacts health utilities during acute infection and long term is important to estimate the full economic impact of SARS-CoV-2 infection. **Methods:** We analyzed EQ-5D-3L survey data from SARS-CoV-2 infected adults (aged ≥16 years) and children (aged 8-15 years) from 3 community and household cohorts in the United States (2020-2022). EQ-5D-3L scores were analyzed at 3 time points after symptom onset or first positive SARS-CoV-2 test result and converted to health utilities on a scale of 0-1 (1 = perfect health). Among adults, regression models were used to compare differences in health utility by demographic/clinical characteristics. **Results:** Among 575 participants with SARS-CoV-2 infections and EQ-5D-3L surveys, mean utilities were nearly 1 throughout the observation period. During days 0-14 after symptom onset, vaccinated participants had higher health utilities (beta, 0.57; 95% CI: 0.07, 1.07). Seeking medical care and having gastrointestinal symptoms (vs none), were associated with lower health utilities (beta, -0.96; 95% CI: −1.60, −0.31; and beta, −0.76; 95% CI: −1.30, −0.21, respectively). During days 15-30 after onset, unemployment was associated with lower health utility (beta, −0.64; 95% CI: −1.15, −0.14). During days 31-90 after onset, underlying conditions were associated with lower health utilities (beta, −0.32; 95% CI: −0.54, −0.09). Results for children were similar to those for adults. **Conclusion:** Mild COVID-19 may have minimal overall impact on quality of life; however, health utilities differed by vaccination status, presence of gastrointestinal symptoms, employment status, and presence of underlying conditions. Vaccination may play an important role in minimizing illness impact from SARS-CoV-2 infection.

## BACKGROUND

Since the first case of COVID-19 in the United States (US) in January 2020,^1^ the COVID-19 pandemic, caused by the SARS-CoV-2 virus, has resulted in over 1 million hospitalizations and deaths in the US^2,3^ Severe cases have significant health and economic impacts,^4,5^ but mild COVID-19 is much more common and can still diminish quality of life, especially for those with persistent symptoms or long COVID.^6–9^ Assessing how SARS-CoV-2 infection affects quality of life, both during acute infection and long-term, is crucial for understanding its overall burden and guidance prevention strategies.

Previous studies of impact of COVID-19 on health-related quality of life have used the EuroQol 5-Dimension Questionnaire (EQ-5D), a standardized generic preference-based instrument used to measure self-rated health,^7,10,11^ and have reported declines in health-related quality of life among nonhospitalized persons with mild illness^12–14^ as well as hospitalized patients with severe illness.^15–17^ We build upon this evidence to estimate health-related quality of life among individuals with asymptomatic to symptomatic, nonhospitalized SARS-CoV-2 infection identified from community and household cohorts with systematic testing for SARS-CoV-2. Using these cohorts, we captured participant health prospectively at enrollment and at defined periods after SARS-CoV-2 infection to calculate health utilities, assess changes from enrollment in self-reported health, and examine differences by demographic and medical characteristics. This is unique to our study design as previous studies estimating health-related quality of life associated with COVID-19 infections were retrospectively conducted. Another unique and important attribute of this study is use of a serial cross-sectional approach to capture health at multiple time points after infection rather than a single time point.

## METHODS

### Source Population

This analysis included data from 3 prospective, longitudinal COVID-19 cohorts within the US followed between 2020 and 2022. The Coronavirus Household Evaluation and Respiratory Testing (C-HEaRT) cohort (N = 1485 people) recruited and followed households with at least 1 child aged <18 years from Salt Lake City, Utah, from August 2020 to August 2021, and New York City, New York, from September 2020 to August 2021.^18^ The Prospective Assessment of COVID-19 in a Community (PACC) cohort (N = 1520 people) randomly sampled and enrolled community members near Marshfield, Wisconsin, and followed participants from November 2020 to July 2022.^19^ The SARS-CoV-2 Epidemiology And Response in Children (SEARCh) cohort (N = 759 people) recruited households with at least 1 child aged <5 years in Maryland and included follow-up from November 2020 to October 2021.^18^ In each cohort, all participants completed an enrollment survey and weekly symptom assessments documenting presence of fever/chills, cough, loss/change in taste/smell, sore throat, muscle/body aches, shortness of breath/difficulty breathing, diarrhea, fatigue, headache, nasal congestion, and vomiting. All cohort participants had weekly self-collected respiratory specimens regardless of reported symptoms. All participants were asked to assess their current health at enrollment and end of follow-up by completing a general health survey consisting of two parts: (1) visual analog scale (VAS; ranking their health on a scale from 0 [worst health imaginable] to 100 [best health imaginable]) and (2) EQ-5D-3L.^10,20^ Responses from EQ-5D-3L were used to compute health utilities, which were assessed for correlation with VAS scores. Participants in the PACC cohort completed the EQ-5D-3L every 6 months as well as the end of the follow-up period. Participants with a respiratory specimen positive for SARS-CoV-2 during the study period were asked to complete the EQ-5D-3L and VAS after the earliest of either symptom resolution (defined as ≥3 days without COVID-19–like illness symptoms) or 4 (PACC cohort) or 5 weeks (C-HeaRT and SEARCh cohorts) for every positive specimen. For C-HeaRT and SEARCh cohorts, EQ-5D-3L/VAS illness follow-up surveys inquired about health during illness. For the PACC cohort, EQ-5D-3L/VAS illness follow-up surveys were administered to collect information about current health. Participants were given the option to complete all data collection surveys online or via telephone.

This study was reviewed and approved by the Institutional Review Boards at the Marshfield Clinic Research Institute (PACC), the University of Utah and Columbia University (C-HEaRT), and Johns Hopkins University (SEARCh). The Centers for Disease Control and Prevention Institutional Review Board reviewed these activities and relied on the approvals of the other participating institutions (see 45 CFR §46; 21 CFR §56).

### Description of EQ-5D-3L Survey and Health Utility

All EQ-5D-3L responses were converted to health utilities at the following time points for further analysis: at enrollment, every 6 months (in the PACC cohort), end of follow-up, and after SARS-CoV-2 infection. Health utilities are values that represent the strength of an individual’s preference for specific health-related outcomes with values ranging from 1 (perfect health) to 0 (being dead); values can also be negative (indicating worse than being dead).^10^ The EQ-5D-3L asks individuals to rate their current health across 5 domains (mobility, self-care, usual activities, pain/discomfort, and anxiety/depression) using a scale from 1 (no problems) to 3 (extreme problems). The completed 5-domain response to the EQ-5D-3L is known as a health state.^20^ Value sets (or preferences) for each health state have been solicited from the general population in various countries.^21–23^ Health utilities for adults (aged ≥16 years) in this study’s cohorts were calculated using previously published US value sets, elicited using the time trade-off valuation method and then mapped to EQ-5D-3L profiles.^21^ As no US value sets existed for children aged 8-15 years, value sets from Spanish children using a time trade-off valuation were used.^24^ No value sets existed for children aged under 8 years old, thus cohort participants aged 4-7 years were excluded from the analysis.

### Demographic/Medical Characteristics

Participants self-reported demographic characteristics at enrollment, including age, sex, race/ethnicity, highest educational level attained, and current employment: employed (full/part-time) and unemployed. Participants also self-reported the presence of select underlying conditions at enrollment, including obesity (derived from self-reported height and weight), mental disorders, lung disease, liver disease, kidney disease, immunocompromised disease, hypertension, heart disease, diabetes, chronic obstructive pulmonary disease (COPD), and cancer. SARS-CoV-2 vaccination status was assessed throughout each cohort’s study period. For C-HEaRT and SEARCh cohorts, vaccination information was self-reported at enrollment and end of follow-up and verified with COVID-19 vaccination cards or data from local immunization information systems or registries. For the PACC cohort, vaccination information was acquired through electronic health records and the Wisconsin Immunization Registry. Individuals’ vaccination status categories were classified by vaccination status at time of their SARS-CoV-2 infection: unvaccinated (no doses of SARS-CoV-2 vaccine), partially vaccinated (1 dose of a 2-dose SARS-CoV-2 vaccine), or fully vaccinated (2 doses of 2 dose SARS-CoV-2 vaccine or 1 dose of 1 dose SARS-CoV-2 vaccine).

For analysis, symptoms were grouped into 8 syndromes: influenza-like illness (fever/chills and cough or sore throat), COVID-like illness (fever/chills, cough, or shortness of breath/difficulty breathing), upper respiratory symptoms (nasal congestion or sore throat), lower respiratory symptoms (cough or shortness of breath/difficulty breathing), neurological symptoms (headache or loss/change in taste/smell), gastrointestinal symptoms (vomiting or diarrhea), constitutional symptoms (fever/chills, muscle/body aches, or fatigue), and moderate COVID-19 symptoms (fever/chills or shortness of breath/difficulty breathing).^25–27^ Individuals could experience one or more syndromes during any illness episode. Surveys after SARS-CoV-2 infection captured whether participants sought medical care for their illness in the 30 days after onset of symptoms or their positive test.

### Statistical Analysis

Data analyses were performed using R (version 4.1.0)^28^ using the package “eq5d.”^29^

### Analytic Population

The analytic population included nonhospitalized participants with a PCR-positive SARS-CoV-2 test result during the study follow-up period, and who had at least 1 completed EQ-5D-3L survey in the 90 days after SARS-CoV-2 infection. Three post-SARS-CoV-2 infection periods were defined for analysis: 0-14 days after onset/positive test (early period), 15-30 days (middle period), and 31-90 days (late period). Participants aged 16 years were categorized as adults and those aged 8-15 years were categorized as children.³ While in many legal and public health frameworks, 16- and 17-year-old individuals are considered as children, the EQ-5D-3L adult version is recommended for those aged 16 years and over,^20^ with health state preferences of older adolescents being more comparable to adults than younger children.^30^

### Health Utility

For each post-SARS-CoV-2 infection period, the distributions of health utilities were examined overall and by demographic or medical characteristics. Correlation between VAS scores and calculated healthy utilities at the same time point was also examined. Among adults, beta regression models were used to assess whether there were differences in health utility by demographic or medical characteristics. A beta distribution was chosen because of its similarities to the distribution of health utilities in which the values range between 0 and 1 (not inclusive) and highly skewed distributions are allowed.^31^ For beta regression, health utility values of 1 were transformed by the formula: Y* = [y(N-1) + 0.5]/N, where Y* is the transformed health utility, y is the original health utility, and N is the sample size,^31^ yielding values of 0.9959. There were no health utilities with a value of 0 in this analysis. Except for models specifically assessing the association between age and health utility, all models were adjusted for age (as a continuous variable). Models assessing education and employment were restricted to adults aged ≥18 years.

### Health Change

The Paretian Classification of Health Change (PCHC)^10^ was used to compare changes in domain-specific responses from a participant over time, comparing each EQ-5D-3L response at enrollment to the response during each infection period. The PCHC categorizes health state changes over time as no change, improve, worsen, or mixed change.^10^ A health state is “improved” if it is better on at least one domain and no worse on any other domain, “worsened” if it is worse in one domain and no better in any other, and “mixed” if it is better in at least one domain, but worse in at least one other.^10^ PCHC analyses were restricted to participants with SARS-CoV-2 infection who completed a survey at enrollment and at least one survey ≤90 days after SARS-CoV-2. For each infection period, the earliest survey after symptom onset/positive test was used in the analysis.

Among adults only, we presented PCHC analyses stratified by demographic/medical characteristics that showed significant associations with health utilities. Fisher exact tests (2-sided) were used to compare the proportion of participants in each health state (*P* <.05 was considered statistically significant).

## RESULTS

### Study Population

**Figure 1.**
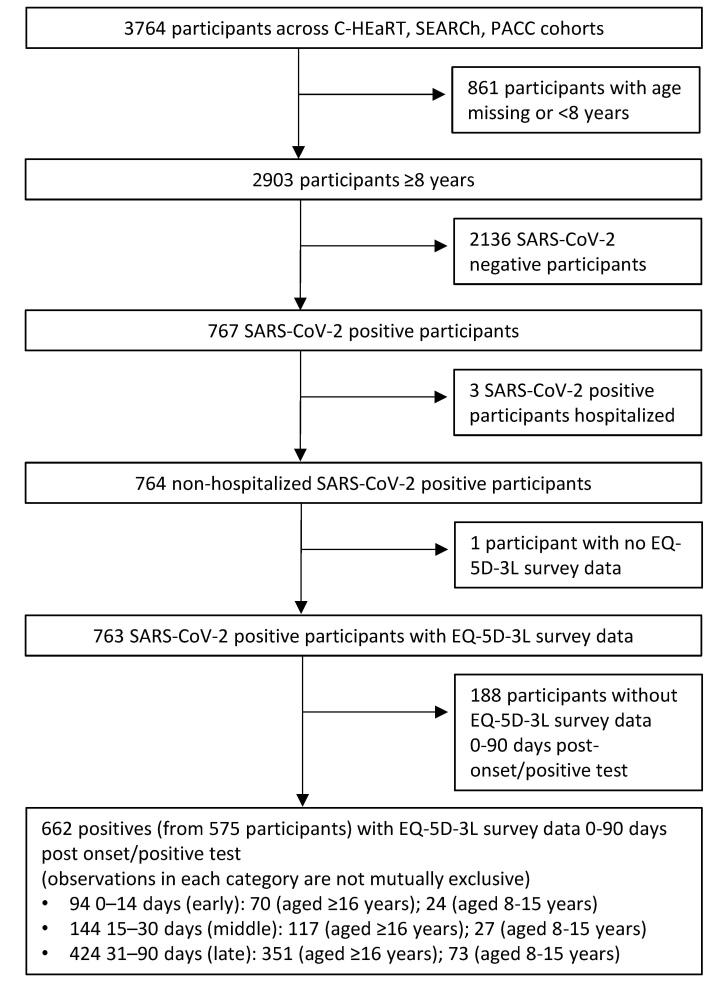
Inclusion and Exclusion of Participants in Analysis of Health-Related Quality of Life from Three Prospective Cohorts in the US Abbreviations: IQR, interquartile range; SD, standard deviation.

Across the 3 cohorts, 3764 participants were enrolled (**Figure 1**). Participants were excluded if they were aged under 8 years or missing age (n = 861), did not test positive for SARS-CoV-2 during the study period (n = 2136), tested positive for SARS-CoV-2 and were hospitalized (n = 3), or with no EQ-5D-3L data within 90 days after symptom onset (n = 1) or positive test for SARS-CoV-2 (n = 188). The final analysis consisted of 575 participants (469 aged ≥16 years and 106 aged 8-15 years) with SARS-CoV-2 infections detected who completed 662 EQ-5D-3L surveys £90 days post-onset/positive test (538 among aged ≥16 years and 124 among aged 8-15 years). Infection dates ranged from October 2020 to June 2022. Out of the 575 participants with at least one SARS-CoV-2 infection, 554 (96%) participants were infected once, and 21 (4%) participants were infected twice. In total, 662 EQ-5D-3L surveys were completed: 94 participants (70 aged ≥16 years and 24 aged 8-15 years) completed the EQ-5D-3L during the early infection period, 144 (117 aged ≥16 years and 27 aged 8-15 years) during the middle infection period, and 424 (351 aged ≥16 years and 73 aged 8-15 years) during the late infection period. Eighty-seven (15%) participants completed the EQ-5D-3L twice within the 90 days after infection (8 during early and middle periods; 26 during early and late periods; and 53 during middle and late periods). No participant completed the EQ-5D-3L survey during all 3 infection periods.

Health utilities reflect preferences for a specific health-related outcome, with values ranging from 1 (perfect health) to 0 (death). SARS-CoV-2 infection stage was defined as the days after symptom onset/positive test that the EQ-5D-3L survey was completed.

Among adults in the analysis (aged ≥16 years), most were from the PACC cohort in Wisconsin, female, aged 16-49 years, non-Hispanic White, college graduates, and currently employed (**Table 1**). Fifty-four percent of the adults with SARS-CoV-2 infection had at least one underlying health condition, most commonly obesity (40%) and hypertension (20%). In the first 30 days following the symptom onset or positive test for SARS-CoV-2, most adults (73%) reported at least one symptom, and 8% sought medical care (**Table 1**). Fifty-five percent of adults were fully vaccinated prior to their SARS-CoV-2 infection. Demographic and medical characteristics of all participants, including children (aged 8-15 years), with SARS-CoV-2 infection are reported in **Supplementary Table S1** and **Table S2**.

**Table 1.** Characteristics of Adult Participants Aged ≥16 Years with SARS-CoV-2 Infection and EQ-5D-3L Survey Data Within 90 Days After Symptom Onset/Positive Test from Three Prospective Cohorts in the US: August 2020–July 2022

	**Periods After Onset/ Positive Test No.(%)^a,b^**			
**Overall^c^**	**0-14 Days (Early SARS-CoV-2 Infection Period)**	**15-30 Days (Middle SARS- CoV-2 Infection Period)**	**31-90 Days (Late SARS- CoV-2 Infection Period)**	
Characteristic	(n = 469)	(n = 70)	(n = 117)	(n = 351)
Location (cohort)				
Maryland (SEARCh)	15 (3.2)	5 (7.1)	10 (8.5)	2 (0.6)
New York (C-HEaRT)	10 (2.1)	5 (7.1)	5 (4.3)	1 (0.3)
Utah (C-HEaRT)	33 (7.0)	16 (22.9)	12 (10.3)	12 (3.4)
Wisconsin (PACC)	411 (87.6)	44 (62.9)	90 (76.9)	336 (95.7)
Sex				
Female	279 (59.5)	43 (61.4)	74 (63.2)	207 (59.0)
Male	190 (40.5)	27 (38.6)	43 (36.8)	144 (41.0)
Age (years)				
Median (IQR)	45.0 (36.0, 61.0)	40.0 (33.0, 57.5)	42.0 (34.0, 55.0)	48.0 (35.0, 63.0)
16-49	266 (56.7)	48 (68.6)	76 (65.0)	189 (53.8)
50-64	109 (23.2)	11 (15.7)	30 (25.6)	84 (23.9)
65+	94 (20.0)	11 (15.7)	11 (9.4)	78 (22.2)
Race/ethnicity				
Asian, non-Hispanic	2 (0.4)	0 (0.0)	2 (1.7)	0 (0.0)
Black, non-Hispanic	3 (0.6)	0 (0.0)	1 (0.9)	3 (0.9)
White, non-Hispanic	443 (94.5)	63 (90.0)	107 (91.5)	339 (96.6)
Multiracial, non-Hispanic	3 (0.6)	1 (1.4)	1 (0.9)	2 (0.6)
Hispanic	18 (3.8)	6 (8.6)	6 (5.1)	7 (2.0)
Education				
Age <18 years	19 (4.1)	4 (5.7)	4 (3.4)	15 (4.3)
Less than high school graduate (adult)	8 (1.7)	1 (1.4)	0 (0.0)	7 (2.0)
High school graduate or equivalent	96 (20.5)	16 (22.9)	19 (16.2)	72 (20.5)
Some college/technical school/associate degree	158 (33.7)	17 (24.3)	45 (38.5)	122 (34.8)
College graduate	186 (39.7)	32 (45.7)	49 (41.9)	133 (37.9)
Employment status				
Age <18 years	19 (4.1)	4 (5.7)	4 (3.4)	15 (4.3)
Employed	325 (69.3)	44 (62.9)	86 (73.5)	246 (70.1)
Unemployed	124 (26.4)	22 (31.4)	27 (23.1)	89 (25.4)
Any underlying condition(s)^d^				
Yes	252 (53.7)	43 (61.4)	57 (48.7)	188 (53.6)
No	217 (46.3)	27 (38.6)	60 (51.3)	163 (46.4)
Obesity				
Yes	189 (40.3)	29 (41.4)	47 (40.2)	145 (41.3)
No	280 (59.7)	41 (58.6)	70 (59.8)	206 (58.7)
Cancer				
Yes	8 (1.7)	1 (1.4)	1 (0.9)	6 (1.7)
No	461 (98.3)	69 (98.6)	116 (99.1)	345 (98.3)
Kidney disease				
Yes	5 (1.1)	0 (0.0)	2 (1.7)	3 (0.9)
No	464 (98.9)	70 (100.0)	115 (98.3)	348 (99.1)
COPD				
Yes	9 (1.9)	1 (1.4)	2 (1.7)	7 (2.0)
No	460 (98.1)	69 (98.6)	115 (98.3)	344 (98.0)
Hypertension				
Yes	95 (20.3)	15 (21.4)	21 (17.9)	73 (20.8)
No	374 (79.7)	55 (78.6)	96 (82.1)	278 (79.2)
Immunocompromised				
Yes	18 (3.8)	4 (5.7)	7 (6.0)	12 (3.4)
No	451 (96.2)	66 (94.3)	110 (94.0)	339 (96.6)
Liver disease				
Yes	1 (0.2)	1 (1.4)	0 (0.0)	0 (0.0)
No	468 (99.8)	69 (98.6)	117 (100.0)	351 (100.0)
Heart disease				
Yes	18 (3.8)	4 (5.7)	3 (2.6)	17 (4.8)
No	451 (96.2)	66 (94.3)	114 (97.4)	334 (95.2)
Mental condition(s)				
Yes	7 (1.5)	3 (4.3)	3 (2.6)	2 (0.6)
No	462 (98.5)	67 (95.7)	114 (97.4)	349 (99.4)
Diabetes				
Yes	29 (6.2)	1 (1.4)	7 (6.0)	25 (7.1)
No	440 (93.8)	69 (98.6)	110 (94.0)	326 (92.9)
Lung disease				
Yes	1 (0.2)	1 (1.4)	0 (0.0)	1 (0.3)
No	468 (99.8)	69 (98.6)	117 (100.0)	350 (99.7)
Characteristics during SARS-CoV-2 infection				
Sought medical care^e^				
Yes, outpatient care	17 (3.6)	9 (12.9)	9 (7.7)	1 (0.3)
Yes, unknown medical care location	19 (4.1)	1 (1.4)	2 (1.7)	17 (4.8)
No	349 (74.4)	60 (85.7)	106 (90.6)	245 (69.8)
COVID-19 vaccination status prior to infection^f^				
Unvaccinated	206 (43.9)	35 (50.0)	57 (48.7)	147 (41.9)
Partially vaccinated	6 (1.3)	0 (0.0)	2 (1.7)	4 (1.1)
Fully vaccinated	257 (54.8)	35 (50.0)	58 (49.6)	200 (57.0)
Self-reported symptom(s)^e,g^				
Yes	341 (72.7)	58 (82.9)	101 (86.3)	240 (68.4)
No	44 (9.4)	12 (17.1)	16 (13.7)	23 (6.6)

Abbreviations: C-HEaRT, Coronavirus Household Evaluation and Respiratory Testing; COPD, chronic obstructive pulmonary disease; IQR, interquartile range; PACC, Prospective Assessment of COVID-19 in a Community; SEARCh, SARS-CoV-2 Epidemiology And Response in Children.

^a^Percentage may not sum to 100 due to missing values.

^b^Unless specified, characteristics represent status at enrollment.

^c^Represents unique number of participants. Participants may be in multiple infection periods.

^d^Self-reported ≥1 of the following: obesity, cancer, kidney disease, COPD, hypertension, immunocompromised, liver disease, heart disease, mental conditions, and diabetes.

^e^Self-reported ≤30 days post-onset/positive test.

^f^Partially vaccinated: Received 1 dose of 2 dose SARS-CoV-2 vaccine; Fully vaccinated: received 2 doses of 2 dose SARS-CoV-2 vaccine or 1 dose of 1 dose SARS-CoV-2 vaccine.

^g^Self-reported ≥1 of the following: fever, cough, loss/change in taste/smell, sore throat, muscle/body aches, shortness of breath, diarrhea, fatigue, headache, nasal congestion, vomiting.

### Health Utilities

**Figure 2.**
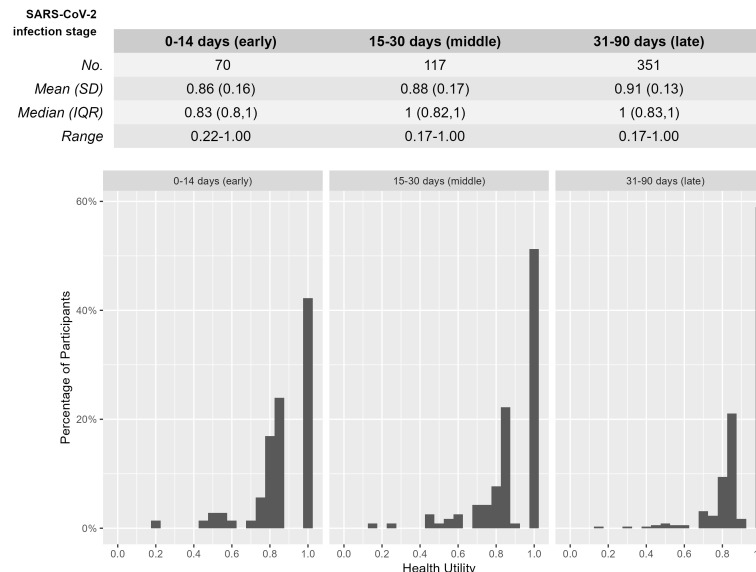
Distribution of Health Utilities Among Adults Aged ≥16 Years with SARS-CoV-2, by Infection Stage Abbreviations: IQR, interquartile range; SD, standard deviation.

Among adults aged ≥16 years, health utilities were skewed towards 1 for all infection periods, with most participants reporting nearly perfect health (**Figure 2**). The mean (SD) health utility during the early infection period (0.86 [0.16]) was lower than during the middle (0.88 [0.17]) and late infection periods (0.91 [0.13]; **Supplementary Table S3**) but these differences were not statistically significant. Among children aged 8-15 years, the mean (SD) health utility did not differ significantly between the early (0.93 [0.11]), middle (0.90 [0.20]) or late (0.95 [0.08]) infection periods. Full descriptions of EQ-5D-3L health states and health utilities in each infection period stratified by age and medical characteristics are presented in **Supplementary Table S3** and **Tables S6-S11**.

Across the two age groups and infection periods, there was a positive correlation between health utilities and reported VAS scores (**Supplementary Figure S1**).

Health utilities reflect preferences for a specific health-related outcome, with values ranging from 1 (perfect health) to 0 (death). SARS-CoV-2 infection stage was defined as the days after symptom onset/positive test that the EQ-5D-3L survey was completed.

### Association Between Health Utilities and Demographic/Medical Characteristics Among Adult Participants

**Figure 3.**
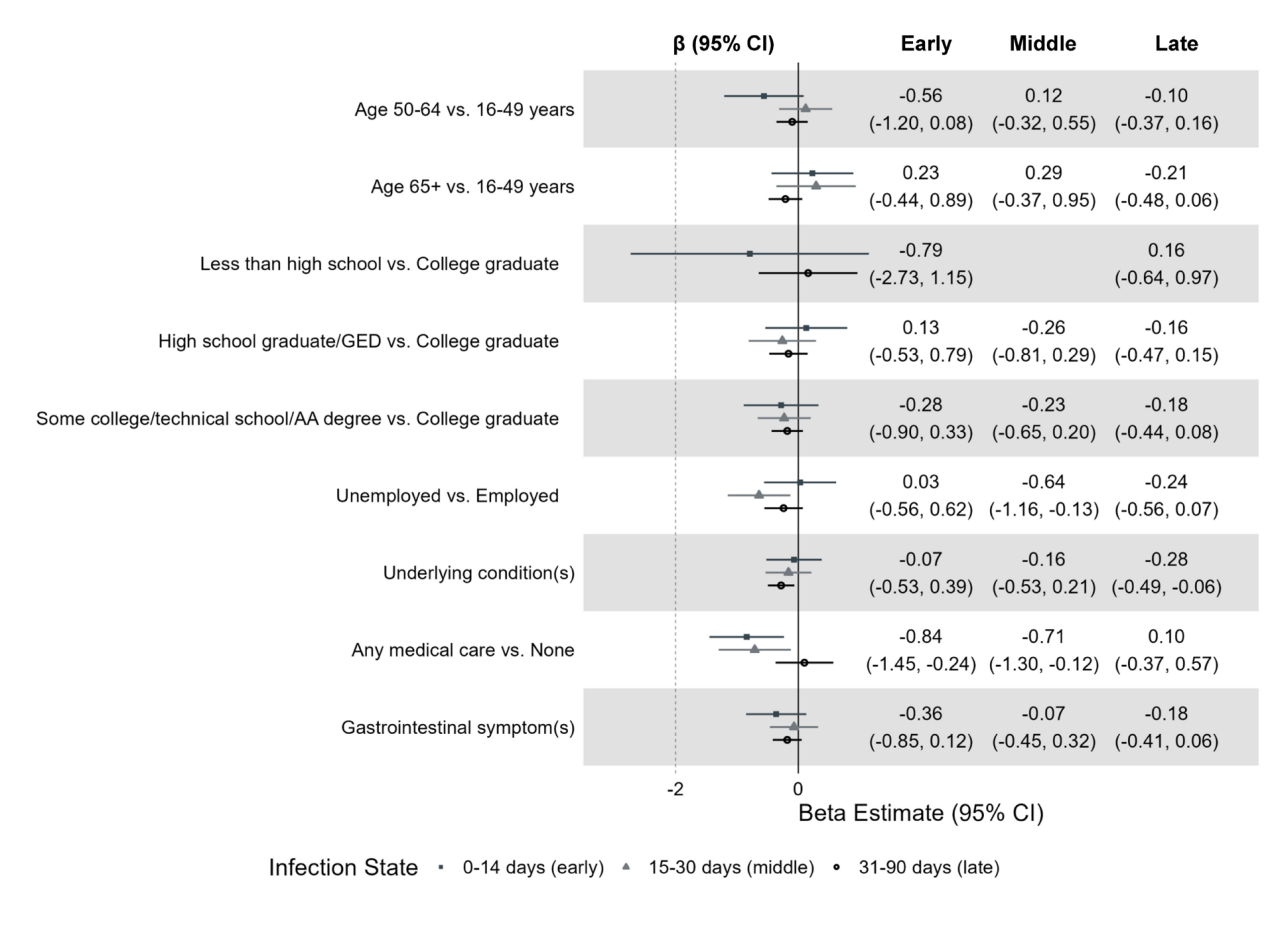
Age-Adjusted Associations Between Health Utility and Demographic/Medical Characteristics Among Adults Aged ≥ 16 Years with SARS-CoV-2 Infection, by Infection Period Abbreviations: AA, associate degree; CI, confidence interval; GED, high school equivalency. SARS-CoV-2 infection periods were defined by the time of the EQ-5D-3L survey after symptom onset/positive test. 95% CIs around beta estimates that cross 0 have *P*  ≥  .05

Using beta regression among adults aged ≥16 years, there was no evidence that health utilities differed by age, sex, education, or COVID-19 syndromes reported during infection (any symptoms, COVID-like illness, influenza-like illness, upper respiratory, lower respiratory, neurological, constitutional, moderate; **Figure 3** and **Supplementary Table S3**). Results for crude and age-adjusted models for the full list of demographics/medical characteristics analyzed are presented as supplemental material (**Supplementary Table S4**).

Among adults who completed an EQ-5D-3L survey during the early infection period (0-14 days after symptom onset or positive test), participants who were fully or partially vaccinated prior to infection had a higher health utility (0.57 higher among those vaccinated) compared with those who were unvaccinated (age-adjusted beta, 0.57; 95% CI: 0.07, 1.07). Participants who self-reported receiving any type of medical care had significantly lower health utility compared with those who did not report seeking medical care (age-adjusted beta, -0.96; 95% CI: -1.60, -0.31). Participants with gastrointestinal symptoms also had significantly lower health utility (age-adjusted beta, -0.76; 95% CI: -1.30, -0.21) (**Figure 3**).

Among adults who completed an EQ-5D-3L survey during the middle infection period, unemployed participants had lower health utility than employed participants (age-adjusted beta, -0.64; 95% CI: -1.15, -0.14) (**Figure 3**).

Among adults who completed an EQ-5D-3L survey during the late infection period, participants with reported underlying conditions had lower health utility than those who did not (age-adjusted beta, -0.32; 95% CI: -0.54, -0.09) (**Figure 3**).

### Change in Health State from Enrollment

**Figure 4.**
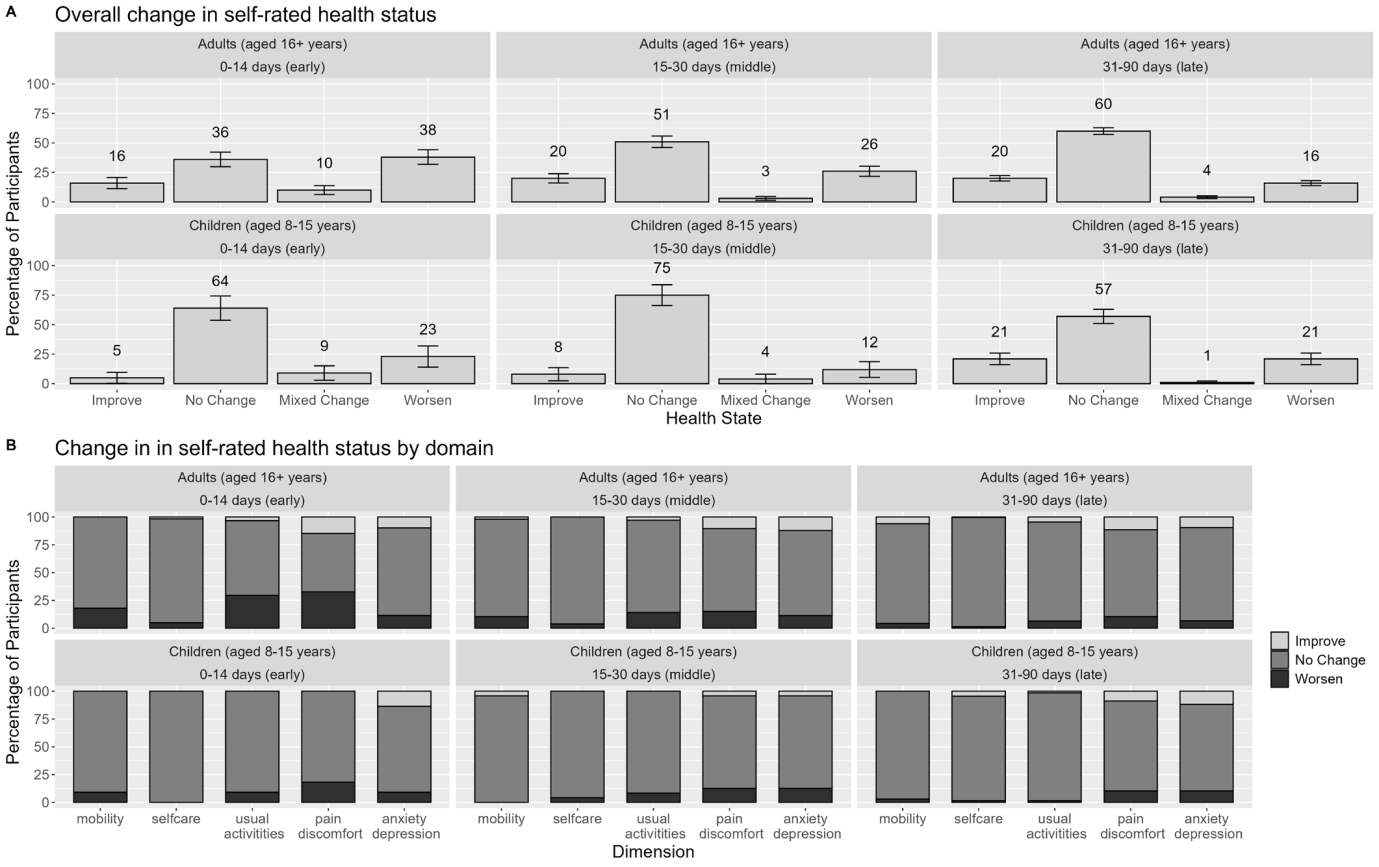
Change in Self-Rated Health Status, Overall (**A**) and by Domain (**B**), from Enrollment Among Participants with SARS-CoV-2 Infection, by Infection Period Among Adults Aged ≥16 Years and Children Aged 8-15 Years

Among participants who completed the EQ-5D-3L survey both at enrollment and during the early infection period (61/70 adults; 22/24 children), 36% of adults and 64% of children reported no change in their health from enrollment to early infection, 16% of adults and 5% of children reported improved health, and 38% of adults and 23% of children reported worsened health (**Figure 4A**). Worsened health was most frequently reported for domains of pain/discomfort (adults, 33%; children,18%) and usual activities (adults, 30%; children, 9%; **Figure 4B**).

Among participants with EQ-5D-3L data at both enrollment and the middle infection period (106/117 adults; 24/27 children), 51% of adults and 75% of children reported no change in their health during infection, 20% of adults and 8% of children reported improved health, and 26% of adults and 12% of children reported worsened health (**Figure 4A**).

Changes among participants with completed EQ-5D-3L surveys at enrollment and ≤90 days after symptom onset/positive test. SARS-CoV-2 infection periods were defined by the time of the EQ-5D-3L survey after symptom onset/positive test.

Among participants with EQ-5D-3L survey at both enrollment and the late infection period (298/351 adults; 68/73 children), 60% of adults and 57% of children reported no change in their health during infection, 20% of adults and 21% of children reported improved health, and 16% of adults and 21% of children reported worsened health (**Figure 4A**).

### Change in Health State from Enrollment by Demographic/Medical Characteristics Among Adults

Among demographic/medical characteristics that showed significant associations with health utilities in the multivariate regression model with sufficient sample size for stratification in each infection period (n > 5) (**Supplementary Table S5**), having gotten vaccinated against COVID-19 prior to infection (fully or partially) was significantly associated with less negative health changes reported during early infection period; 54% of participants (14/26) who were unvaccinated prior to their infection reported health was worsened vs. 26% (9/35) of vaccinated participants (*P* < .01).

## DISCUSSION

Using data from 3 prospective cohorts in the US, health utilities were calculated for 3 post-SARS-CoV-2 infection periods £90 days post–symptom onset/positive test. Compared with reported health at cohort enrollment, 38% of adults aged ≥16 years and 23% of children aged 8-15 years with SARS-CoV-2 experienced worsened health in the first 2 weeks after symptom onset or first positive test. Worsened health was most commonly due to pain/discomfort for all participants and having a harder time performing usual activities among adults. There was evidence that adults who were vaccinated with at least 1 dose against SARS-CoV-2 had improved health during the first 2 weeks of infection compared with those who were unvaccinated.

Most of the infections in the cohorts were symptomatic but mild, with only 8% reporting seeking medical care during their illness, and most reporting no problems with their health during all infection periods. This outcome may be partially explained by the demographic composition of our sample. Despite over half the sample having underlying conditions (53.7%), the minority of participants did not have other risk factors for severe COVID-19 and hospitalization (aged >65 years, certain races and ethnicities, and being unvaccinated against SARS-CoV-2).^32,33^ However, there was a sizeable proportion of adult participants who reported a worsening in health during SARS-CoV-2 infection, most commonly due to pain/discomfort. Problems were more frequently reported during the first 2 weeks after infection compared with later periods of infection. Our findings were consistent with other EQ-5D studies of patients with mild to severe COVID-19, where problems due to pain/discomfort during illness compared with the other domains (mobility, self-care, usual activities, anxiety/depression) are most frequently reported.^13,34,35^ “COVID-pain” has been described by previous studies as a vast spectrum of clinical manifestations of the COVID-19 illness.^31^ The characterization includes myalgia, joint pain, sore throat, abdominal pain, chest pain, and headache. These are common symptoms associated with acute infection and may be similar to what was observed in these cohorts.^36,37^

Consistent with existing literature exploring impact of SARS-CoV-2 infection among children, health remained largely unaffected among children in our study. Another EQ-5D study among children with SARS-CoV-2 infection found that most children with persistent symptoms reported mild or no impairment in their quality of life, including school function, 3 to 6 months after infection.^38^ In this study, the proportion of children with worsened health over time was approximately half of the proportion of adults who reported worsened health. While children and adults have similar incidence rates of SARS-CoV-2 infection,^32^ children tend to have milder symptoms or be asymptomatic.^39,40^ Differences in immune response, tobacco use, alcohol consumption, and comorbidities have been proposed explanations for illness severity gaps between adults and children.^41,42^

Despite overall high health utilities reported among these cohorts, there was variation when stratified by demographic and medical characteristics at different postinfection periods. Vaccination against SARS-CoV-2 had a positive effect on health utility among adults during the first 14 days after symptom onset/positive test. Our findings are consistent with other studies conducted among those with SARS-CoV-2 infection, which observed increased health utility and VAS scores among participants vaccinated against SARS-CoV-2 compared with those unvaccinated.^43,44^ Vaccinated individuals have also been shown to have less of a health decline, via health utility and VAS scores, from enrollment to up to 4 weeks post-positive RT-PCR test compared with unvaccinated counterparts.^43^ Similarly, vaccinated participants were less likely to report worsened health from enrollment to ≤14 days post-symptom onset/positive test.

Adults who sought medical care during their illness and those with gastrointestinal symptoms had significantly worse self-reported health during the first 2 weeks of infection. These findings support one another, given that individuals who seek medical care generally have more severe symptoms.^45^ However, these risk factors were transient and not significantly related to worse health in later weeks after infection, as we defined them in this study at 31-90 days. Unemployed adults had lower health utility in the subsequent 2 weeks after infection. Other studies have reported increased illness severity and ICU admittance among unemployed adults with COVID-19.^46,47^ Not only do unemployed individuals have limited access to medical care/resources,^48^ but they also have been suggested to be older, a main risk factor for severe illness.^46^ Lower health utilities in the months after infection, in this study, were only associated with having underlying conditions. Other studies have also observed that having underlying conditions can increase the risk of lingering symptoms or developing post/long COVID.^49^

This study combined data from 3 large cohorts from different US locations followed during similar timeline and with comparable methods, including standardized implementation of the EQ-5D-3L survey to measure health utility and the VAS to measure self-reported health. A further strength of the study was the large number of SARS-CoV-2 infections that were detected. Because we collected EQ-5D-3L data at multiple time points, we were able to observe changes in self-rated health over time. However, at least 4 limitations should be considered when interpreting our findings. First, even with a large number of SARS-CoV-2 infections, the cohorts had limited diversity in some of the demographic and medical characteristics, which limited our ability to fully adjust for potential confounding, and participants may not be representative of the general population in the US. With a relatively young and healthy sample, our estimates likely underestimate health-related quality-of-life impact of SARS-CoV-2 infection. Relatedly, we were only able to present limited stratified results for participants aged 8 to 15 years due to small numbers. Second, we were unable to conduct time series analysis because few participants completed more than one EQ-5D-3L survey after illness or infection onset. Third, the EQ-5D-3L surveys in the C-HEaRT and SEARCh cohorts conducted after an infection asked about health during the illness. This is different from the wording used in the PACC cohort, which asked about “current health” within the EQ-5D-3L survey completed after the acute phase of infection. This methodologic difference could bias the early infection health utilities from C-HEaRT and SEARCh and may not be representative of convalescent health. We do not believe this difference had an impact on EQ-5D-3L surveys completed during the middle or late infection period. Fourth, results regarding vaccination did not consider time since vaccination, an important determinant of effectiveness. Fifth, value sets for health utilities for US children, in general, and for all children aged under 8 years were not available. We used value sets derived from a Spanish population of children, aged 8 to 15 years, as a proxy, which may not represent US children due to cultural differences and preferences.

## CONCLUSIONS

Among participants infected with SARS-CoV-2 across 3 cohorts followed from August 2020 to July 2022, self-rated health at various times during 90 days after infection onset infection was lower in those who sought medical care, had gastrointestinal symptoms, were unemployed, and had underlying health conditions. Vaccinated participants were less likely to experience a health decline during the first two weeks following infection than unvaccinated participants. Overall, health-related quality of life measured by health utilities, VAS scores, and Paretian Classification of Health Change was largely unaffected by nonhospitalized SARS-CoV-2 infection. Although health utilities were impacted most during the first 2 weeks of infection, population-level impacts are high given the high infection rates during peak circulation of SARS-CoV-2 viruses. Data from this study may support future evaluations of the full burden of SARS-CoV-2 infection and benefits of interventions, such as vaccination.

### Disclosures

H.Q.N. reports support for unrelated work from CSL Seqirus, ModernaTX, and GSK. M.S.S. reports support, paid to Trustees of Columbia, from CDC for grants related to SARS-CoV-2 and COVID-19 vaccination, from NIH for grant related to long COVID, and from American Academy of Pediatrics. M.H. reports support for unrelated work from Pfizer. M.D.K. reports support for unrelated work from the Coalition for Epidemic Preparedness Innovations, the Gates Foundation, the World Health Organization, the National Institute of Dental and Craniofacial Research, Pfizer and Merck. All other authors report no conflicts of interest. The findings and conclusions in this report are those of the authors and do not necessarily represent the official position of the Centers for Disease Control and Prevention.

## Supplementary Material

Online Supplementary Material
