# Supplementary material for: Estimating Impact of SARS-CoV-2 Infection on Health-Related Quality of Life Among Persons Aged 8 Years and Older, August 2020–July 2022
Source: J Health Econ Outcomes Res. 2026 Jul 28;13(2):16–26. doi: 10.36469/001c.163131 (PMC13423352; doi:10.36469/001c.163131)
Supplement: Online Supplementary Material [file jheor_2026_13_2_163131_355841.pdf]

Journal of Health Economics and Outcomes Research

Online Supplementary Material

Estimating Impact of SARS-CoV-2 Infection on Health-Related Quality of Life Among Persons Aged 8 Years and Older, August 2020–July 2022. *JHEOR*. 2026;13(2):16-26. doi:10.36469/jheor.2026.163131

Table S1: Characteristics of Participants Aged ≥8 Years with SARS-CoV-2 Infection with and without EQ-5D Survey Data Within 90 Days After Symptom Onset/Positive Test from three Prospective, Longitudinal Cohorts in the United States from August 2020--July 2022

Table S2: Characteristics of Adult Participants Aged ≥8 Years with SARS-CoV-2 Infection and EQ-5D Survey Data Within 90 Days After Symptom Onset/Positive Test from three Prospective, Longitudinal Cohorts in the United States from August 2020-July 2022

Table S3: Descriptive Statistics for Health Utilities Among Participants Aged ≥8 Years with SARS-CoV-2 Infection, by Infection Period Stratified by Age

Table S4: Associations Between Health Utility and Demographic/Medical Chracteristics Among Adults Aged ≥16 Years

Table S5: Change in Overall Self-Rated Health Status from Enrollment Among Adults with SARS-CoV-2 Infection, by Infection Period, Stratified by Demographic/Medical Characteristics with >5 Participants in Each Infection Period

Table S6: Descriptive Statistics for Health Utilities Among Participants Aged ≥8 Years with SARS-CoV-2 Infection, Stratified by Age and Reported Presence of Symptoms (Restricted to 15-30 Day and 31-90 Day Infection Periods

Table S7: Descriptive Statistics for Health Utilities Among Participants Aged ≥8 Years with SARS-CoV-2 Infection, by Infection Period, Stratified byAge, Presence of Symptoms, and Vaccination Status Prior to Infection (Restricted to 15-30 Day and 31-90 Day Infection Periods)

This supplementary material has been provided by the authors to give readers additional information about their work.

This is an open-access article distributed under the terms of the Creative Commons Attribution 4.0 International License (CCBY-4.0). View this license's legal deed at http://creativecommons.org/licenses/by/4.0 and legal code at http://creativecommons.org/licenses/by/4.0/legalcode for more information.

Supplemental Table 1. Characteristics of participants aged ≥8 years with SARS-CoV-2 infection with and without EQ-5D survey data within 90 days after symptom onset/positive test from three prospective, longitudinal cohorts in the United States from August 2020-July 2022

	Periods after onset/ positive test No.(%)[a,b]				
	Participants with SARS-CoV-2 infection[c]	Participants with SARS-CoV-2 infection without EQ-5D data within 90 days after infection	Participants with SARS-CoV-2 infection with EQ-5D data during 0-14 days after infection (early)	Participants with SARS-CoV-2 infection with EQ-5D data during 15-30 days after infection (middle)	Participants with SARS-CoV-2 infection with EQ-5D data during 31-90 days after infection (late)
No.	(n=763)	(n=188)	(n=94)	(n=144)	(n=424)
Location (Cohort)					
Maryland (SEARCh)	24 (3.1)	7 (3.7)	6 (6.4)	11 (7.6)	2 (0.5)
New York (C-HEaRT)	21 (2.8)	8 (4.3)	6 (6.4)	7 (4.9)	1 (0.2)
Utah (C-HEaRT)	66 (8.7)	24 (12.8)	22 (23.4)	15 (10.4)	14 (3.3)
Wisconsin (PACC)	652 (85.5)	149 (79.3)	60 (63.8)	111 (77.1)	407 (96.0)
Sex					
Female	433 (56.7)	102 (54.3)	55 (58.5)	88 (61.1)	242 (57.1)
Male	330 (43.3)	86 (45.7)	39 (41.5)	56 (38.9)	182 (42.9)
Age (in years)					
Median (IQR)	40.00 (24.00, 59.00)	40.00 (18.75, 61.00)	34.50 (15.25, 45.75)	38.00 (25.00, 52.00)	41.50 (27.00, 59.00)
8-15	147 (19.3)	41 (21.8)	24 (25.5)	27 (18.8)	73 (17.2)
16-49	338 (44.3)	72 (38.3)	48 (51.1)	76 (52.8)	189 (44.6)
50-64	140 (18.3)	31 (16.5)	11 (11.7)	30 (20.8)	84 (19.8)
65+	138 (18.1)	44 (23.4)	11 (11.7)	11 (7.6)	78 (18.4)
Race/Ethnicity					
Asian, non-Hispanic	3 (0.4)	0 (0.0)	1 (1.1)	2 (1.4)	0 (0.0)
Black, non-Hispanic	4 (0.5)	1 (0.5)	0 (0.0)	1 (0.7)	3 (0.7)
White, non-Hispanic	705 (92.4)	165 (87.8)	84 (89.4)	130 (90.3)	409 (96.5)
Multiracial, non-Hispanic	8 (1.0)	2 (1.1)	2 (2.1)	2 (1.4)	3 (0.7)
Hispanic	42 (5.5)	19 (10.1)	7 (7.4)	9 (6.2)	9 (2.1)
Unknown race	1 (0.1)	1 (0.5)	0 (0.0)	0 (0.0)	0 (0.0)
Education					
Age <18 years	169 (22.1)	46 (24.5)	28 (29.8)	30 (20.8)	87 (20.5)
Less than high school	250 (32.8)	64 (34.0)	32 (34.0)	49 (34.0)	133 (31.4)
High school graduate/GED	14 (1.8)	6 (3.2)	1 (1.1)	0 (0.0)	7 (1.7)
Some college/technical school/AA degree	130 (17.0)	34 (18.1)	16 (17.0)	19 (13.2)	72 (17.0)
College graduate	195 (25.6)	37 (19.7)	17 (18.1)	45 (31.2)	122 (28.8)
Employment status					
Age <18 years	169 (22.1)	46 (24.5)	28 (29.8)	30 (20.8)	87 (20.5)
Unemployed	177 (23.2)	53 (28.2)	22 (23.4)	27 (18.8)	89 (21.0)
Employed	414 (54.3)	89 (47.3)	44 (46.8)	86 (59.7)	246 (58.0)
Underlying condition(s) [d]					
Yes	353 (46.3)	87 (46.3)	46 (48.9)	60 (41.7)	196 (46.2)
No	410 (53.7)	101 (53.7)	48 (51.1)	84 (58.3)	228 (53.8)
COVID-19 vaccination status prior to infection[e]					
Unvaccinated	352 (46.1)	86 (45.7)	51 (54.3)	77 (53.5)	184 (43.4)
Partially vaccinated	12 (1.6)	1 (0.5)	1 (1.1)	3 (2.1)	7 (1.7)
Fully vaccinated	388 (50.9)	90 (47.9)	42 (44.7)	64 (44.4)	233 (55.0)
Self-reported symptom(s) ≤30 days post-onset/positive test[f]					
Yes	507 (66.4)	71 (37.8)	78 (83.0)	122 (84.7)	297 (70.0)
No	92 (12.1)	31 (16.5)	16 (17.0)	22 (15.3)	29 (6.8)
Days between symptom onset/positive test and EQ-5D-3L/ VAS completion					
Mean (SD)	37.85 (22.22)	—	8.34 (3.94)	23.40 (4.96)	52.45 (17.44)
Median (IQR)	34.00 (21.00, 53.50)	—	8.50 (6.00, 12.00)	23.00 (20.00, 28.00)	50.00 (36.00, 66.00)

[a] Percentage may not sum to 100 due to missing values
[b] Unless specified, characteristics represent enrollment status
[c] Represents unique number of participants. Participants may be in multiple infection periods
[d] Self-reported at least one of the following: obesity, cancer, kidney disease, COPD, hypertension, immunocompromised, liver disease, heart disease, mental conditions, and diabetes
[e] Partially vaccinated: received 1 dose of 2 dose SARS-CoV-2 vaccine; Fully vaccinated: received 2 doses of 2 dose SARS-CoV-2 vaccine or 1 dose of 1 dose SARS-CoV-2 vaccine
[f] Self-reported at least one of the following: fever, cough, loss/change in taste/smell, sore throat, muscle/body aches, shortness of breath, diarrhea, fatigue, headache, nasal congestion, vomiting

Supplemental Table 2. Characteristics of adult participants aged ≥8 years with SARS-CoV-2 infection and EQ-5D-3L survey data within 90 days after symptom onset/positive test from three prospective, longitudinal cohorts in the United States from August 2020-July 2022

	Cohort No.(%)[a,b,c]		
Characteristic	C-HEaRT (n=55)	PACC (n=503)	SEARCh (n=17)
Sex			
Female	32 (58.2)	289 (57.5)	10 (58.8)
Male	23 (41.8)	214 (42.5)	7 (41.2)
Age (in years)			
Median (IQR)	33.00 (16.5, 41.0)	42.00 (27.0, 60.0)	35.00 (32.00, 38.00)
08-15	12 (21.8)	92 (18.3)	2 (11.8)
16-49	41 (74.5)	210 (41.7)	15 (88.2)
50-64	2 (3.6)	107 (21.3)	0 (0.0)
65+	0 (0.0)	94 (18.7)	0 (0.0)
Race/Ethnicity			
Asian, non-Hispanic	0 (0.0)	1 (0.2)	2 (11.8)
Black, non-Hispanic	1 (1.8)	1 (0.2)	1 (5.9)
White, non-Hispanic	41 (74.5)	488 (97.0)	11 (64.7)
Multiracial, non-Hispanic	0 (0.0)	3 (0.6)	3 (17.6)
Hispanic	13 (23.6)	10 (2.0)	0 (0.0)
Education			
Age <18 years	16 (29.1)	105 (20.9)	2 (11.8)
Less than high school (adult)	1 (1.8)	7 (1.4)	0 (0.0)
High school grad/GED	6 (10.9)	87 (17.3)	3 (17.6)
Some college/technical school/AA degree	7 (12.7)	149 (29.6)	2 (11.8)
College grad	25 (45.5)	151 (30.0)	10 (58.8)
Employment Status			
Age <18 years	16 (29.1)	105 (20.9)	2 (11.8)
Employed	28 (50.9)	284 (56.5)	13 (76.5)
Unemployed	11 (20.0)	111 (22.1)	2 (11.8)
Any underlying condition(s)[d]			
Yes	15 (27.3)	241 (47.9)	10 (58.8)
No	40 (72.7)	262 (52.1)	7 (41.2)
Sought medical care[e]			
Yes, Outpatient care	9 (16.4)	0 (0.0)	11 (64.7)
Yes, unknown medical care location	1 (1.8)	19 (3.8)	0 (0.0)
No	45 (81.8)	390 (77.5)	6 (35.3)
COVID-19 vaccination status prior to infection			
Unvaccinated	42 (76.4)	212 (42.1)	14 (82.4)
Partially vaccinated	2 (3.6)	7 (1.4)	1 (5.9)
Fully vaccinated	11 (20.0)	284 (56.5)	2 (11.8)
Self-reported symptom(s)[e,g]			
Yes	47 (85.5)	360 (71.6)	16 (94.1)
No	8 (14.5)	49 (9.7)	1 (5.9)

[a] Percentage may not sum to 100 due to missing values
[b] Unless specified, characteristics represent status at enrollment
[c] Represents unique number of participants.
[d] Self-reported at least one of the following: obesity, cancer, kidney disease, COPD, hypertension, immunocompromised, liver disease, heart disease, mental conditions, and diabetes
[e] Self-reported ≤30 days post-onset/positive test
[f] Partially vaccinated: received 1 dose of 2 dose SARS-CoV-2 vaccine; Fully vaccinated: received 2 doses of 2 dose SARS-CoV-2 vaccine or 1 dose of 1 dose SARS-CoV-2 vaccine
[g] Self-reported at least one of the following: fever, cough, loss/change in taste/smell, sore throat, muscle/body aches, shortness of breath, diarrhea, fatigue, headache, nasal congestion, vomiting

Supplemental Table 3. Descriptive statistics for health utilities among participants aged ≥8 years with SARS-CoV-2 infection, by infection period stratified by age

		Participants ≥16 years[a]			
SARS CoV-2 infection period	No.	Mean (SD)	Median (IQR)	Bootstrapped 95% CI	Range
Overall	538	0.90 (0.14)	1.00 (0.83, 1.00)	0.88,0.91	0.17,1.00
0-14 days (early)	70	0.86 (0.16)	0.83 (0.80, 1.00)	0.82,0.89	0.22,1.00
15-30 days (middle)	117	0.88 (0.17)	1.00 (0.82, 1.00)	0.85,0.9	0.17,1.00
31-90 days (late)	351	0.91 (0.13)	1.00 (0.83, 1.00)	0.9,0.93	0.17,1.00
		Participants 8-15 years[b]			
SARS CoV-2 infection period	No.	Mean (SD)	Median (IQR)	Bootstrapped 95% CI	Range
Overall	124	0.94 (0.12)	1.00 (0.89, 1.00)	0.91,0.96	0.12,1.00
0-14 days (early)	24	0.93 (0.11)	1.00 (0.89, 1.00)	0.88,0.97	0.61,1.00
15-30 days (middle)	27	0.90 (0.20)	1.00 (0.89, 1.00)	0.82,0.97	0.12,1.00
31-90 days (late)	73	0.95 (0.08)	1.00 (0.89, 1.00)	0.93,0.97	0.71,1.00

SD = standard deviation; IQR = interquartile range; Cl = confidence interval
SARS-CoV-2 infection period = defined by the time of EQ-5D-3L survey after symptom onset/positive test
[a] US value sets for adults (aged ≥16 years) (Shaw JW, Johnson JA, Coons SJ. US valuation of the EQ-5D health states: development and testing of the D1 valuation model. *Med Care*. Mar 2005;43(3):203-20. doi:10.1097/00005650-200503000-00003)
[b] Value sets used to calculate health utilities derived from Spanish children (aged 8-15 years) onset/positive test (Ramos-Goñi JM, Oppe M, Estévez-Carrillo A, et al. Accounting for Unobservable Preference Heterogeneity and Evaluating Alternative Anchoring Approaches to Estimate Country-Specific EQ-5D-Y Value Sets: A Case Study Using Spanish Preference Data. *Value in Health*. 2022;25(5):835-843. doi:10.1016/j.jval.2021.10.013)

Supplemental Table 4. Associations between health utility and demographic/medical characteristics among adults aged ≥16 years

	0-14 days (early)[a]		15-30 days (middle)[a]		31-90 days (late)[a]	
	Crude[b]	Age-adjusted[b]	Crude[b]	Age-adjusted[b]	Crude[b]	Age-adjusted[b]
Demographic/medical characteristics	β (95% CI)	β (95% CI)	β (95% CI)	β (95% CI)	β (95% CI)	β (95% CI)
Age 50-64 vs. 16-49 years	-0.55 (-1.18, 0.09)	--	0.12 (-0.32, 0.55)	--	-0.10 (-0.37, 0.16)	--
Age 65+ vs. 16-49 years	0.23 (-0.43, 0.89)	--	0.29 (-0.36, 0.94)	--	-0.21 (-0.48, 0.06)	--
Sex (Male vs. Female)	0.26 (-0.22, 0.74)	0.26 (-0.23, 0.74)	0.11 (-0.28, 0.50)	0.08 (-0.31, 0.48)	-0.02 (-0.24, 0.20)	-0.01 (-0.23, 0.21)
Black, non-Hispanic vs. White, non-Hispanic[c]	--	--	**-2.73 (-4.61, -0.84)**	**-2.64 (-4.53, -0.75)**	0.52 (-0.65, 1.69)	0.49 (-0.68, 1.66)
Asian, non-Hispanic vs. White, non-Hispanic	--	--	0.62 (-0.82, 2.06)	0.67 (-0.77, 2.11)	--	--
Multiracial, non-Hispanic vs. White, non-Hispanic[c]	**-2.06 (-3.81, -0.31)**	**-2.08 (-3.86, -0.29)**	0.62 (-1.41, 2.64)	0.76 (-1.29, 2.80)	0.52 (-0.91, 1.95)	0.48 (-0.95, 1.91)
Hispanic vs. White, non-Hispanic	-0.20 (-1.01, 0.62)	-0.20 (-1.03, 0.63)	-0.37 (-1.20, 0.46)	-0.33 (-1.16, 0.51)	0.20 (-0.57, 0.97)	0.19 (-0.58, 0.96)
Less than high school vs. College graduate[d]	-0.70 (-2.60, 1.19)	-0.76 (-2.66, 1.15)	--	--	0.06 (-0.72, 0.84)	0.16 (-0.64, 0.96)
High school graduate/GED vs. College graduate[d]	0.18 (-0.43, 0.78)	0.13 (-0.52, 0.78)	-0.19 (-0.73, 0.36)	-0.26 (-0.81, 0.29)	-0.21 (-0.51, 0.08)	-0.15 (-0.46, 0.15)
Some college/technical school/AA degree vs. College graduate[d]	-0.24 (-0.82, 0.34)	-0.28 (-0.89, 0.32)	-0.15 (-0.57, 0.27)	-0.22 (-0.65, 0.20)	-0.21 (-0.46, 0.04)	-0.18 (-0.44, 0.08)
Unemployed vs. Employed[d]	0.09 (-0.42, 0.60)	0.04 (-0.55, 0.62)	-0.33 (-0.78, 0.11)	**-0.64 (-1.15, -0.14)**	-0.26 (-0.51, -0.01)	-0.24 (-0.55, 0.07)
Underlying condition(s)	-0.17 (-0.65, 0.31)	-0.24 (-0.75, 0.26)	-0.17 (-0.54, 0.20)	-0.21 (-0.59, 0.17)	**-0.33 (-0.55, -0.12)**	**-0.32 (-0.54, -0.09)**
Vaccination status prior to infection (Fully/Partially vs. Unvaccinated)[e]	**0.53 (0.07, 1.00)**	**0.57 (0.07, 1.07)**	0.24 (-0.13, 0.61)	0.24 (-0.14, 0.61)	0.04 (-0.18, 0.26)	
Any medical care vs. None	**-0.94 (-1.57, -0.31)**	**-0.96 (-1.60, -0.31)**	-0.47 (-1.11, 0.16)	-0.43 (-1.07, 0.22)	0.12 (-0.37, 0.60)	0.09 (-0.40, 0.58)
Any symptom(s)[f]	-0.23 (-0.85, 0.40)	-0.22 (-0.85, 0.40)	-0.02 (-0.56, 0.52)	0.04 (-0.52, 0.59)	0.10 (-0.13, 0.33)	0.09 (-0.14, 0.32)
ILI symptom(s)[f]	-0.38 (-0.86, 0.10)	-0.38 (-0.86, 0.11)	-0.34 (-0.71, 0.03)	-0.32 (-0.70, 0.06)	-0.05 (-0.27, 0.18)	-0.07 (-0.29, 0.16)
CLI symptom(s)[f]	-0.32 (-0.83, 0.19)	-0.33 (-0.84, 0.18)	-0.26 (-0.67, 0.16)	-0.23 (-0.66, 0.21)	0.01 (-0.21, 0.23)	0.00 (-0.22, 0.22)
Constitutional symptom(s)[f]	-0.42 (-0.95, 0.11)	-0.42 (-0.95, 0.11)	-0.30 (-0.72, 0.11)	-0.28 (-0.70, 0.13)	0.02 (-0.20, 0.23)	0.01 (-0.21, 0.23)
Moderate symptoms(s)[f]	-0.43 (-0.89, 0.04)	-0.43 (-0.90, 0.04)	**-0.38 (-0.76, -0.01)**	-0.36 (-0.75, 0.02)	-0.06 (-0.28, 0.17)	-0.07 (-0.29, 0.15)
Upper respiratory symptom(s)[f]	-0.10 (-0.62, 0.41)	-0.11 (-0.63, 0.41)	-0.10 (-0.58, 0.38)	-0.08 (-0.56, 0.40)	-0.00 (-0.22, 0.22)	-0.01 (-0.24, 0.21)
Lower respiratory symptom(s)[f]	-0.33 (-0.82, 0.16)	-0.35 (-0.84, 0.14)	-0.26 (-0.66, 0.15)	-0.23 (-0.65, 0.19)	0.00 (-0.21, 0.22)	-0.00 (-0.22, 0.21)
Neurological symptom(s)[f]	-0.20 (-0.69, 0.30)	-0.19 (-0.70, 0.31)	-0.28 (-0.68, 0.13)	-0.24 (-0.68, 0.19)	0.03 (-0.18, 0.25)	0.02 (-0.20, 0.24)
Gastrointestinal symptom(s)[f]	**-0.74 (-1.29, -0.19)**	**-0.76 (-1.30, -0.21)**	-0.09 (-0.51, 0.33)	-0.05 (-0.48, 0.37)	-0.16 (-0.42, 0.11)	-0.16 (-0.42, 0.11)

CI = Confidence Interval, AA = Associates of Arts, GED = General Educational Development, ILI = Influenza-like illness, CLI = COVID-like illness;
SARS-CoV-2 infection period = defined by the time of EQ-5D-3L survey after symptom onset/positive test
Adults = participants aged ≥16 years
[a] SARS-CoV-2 infection period observations not mutually exclusive
[b] Boldface indicates statistically significant results at the 0.05 level
[c] Significant findings may be underpowered due to small sample size
[d] Models restricted to participants aged ≥18 years
[e] Partially vaccinated: received 1 dose of 2 dose SARS-CoV-2 vaccine; Fully vaccinated: received 2 doses of 2 dose SARS-CoV-2 vaccine or 1 dose of 1 dose SARS-CoV-2 vaccine
[f] Referent group are participants who did not meet syndrome definition

Supplemental Table 5. Change in overall self-rated health status from enrollment among adults with SARS-CoV-2 infection, by infection period stratified by demographic/medical characteristics with >5 participants in each infection period

**0–14-days (early) (n=61)[a]**			
	Vaccinated (Fully/Partially) n (column %)	Unvaccinated n (column %)	p-value[b]
No Change	13 (37.1)	9 (34.6)	**<0.01**
Improve	10 (28.6)	0 (0.0)	
Worsen	9 (25.7)	14 (53.8)	
Mixed Change	3 (8.6)	3 (11.5)	
	Reported seeking medical care during SARS-CoV-2 infection n (column %)	Reported seeking no medical care during SARS-CoV-2 infection n (column %)	p-value[b]
No Change	1 (12.5)	19 (37.3)	0.22
Improve	0 (0.0)	10 (19.6)	
Worsen	7 (87.5)	17 (33.3)	
Mixed Change	0 (0.0)	5 (9.8)	
	Reported gastrointestinal symptoms n (column %)	Reported no gastrointestinal symptoms n (column %)	p-value[b]
No Change	2 (14.3)	20 (42.6)	0.06
Improve	1 (7.1)	9 (19.1)	
Worsen	9 (19.1)	14 (29.8)	
Mixed Change	2 (14.3)	4 (8.5)	
**15–30 days (middle) (n=106)[a]**			
	Employed n (column %)	Unemployed n (column %)	p-value[b]
No Change	44 (56.4)	7 (29.2)	0.07
Improve	12 (15.4)	8 (33.3)	
Worsen	20 (25.6)	8 (33.3)	
Mixed Change	2 (2.6)	1 (4.2)	
**31–90 days (late) (n=298)[a]**			
	Reported underlying condition(s) n (column %)	Reported no underlying condition(s) n (column %)	p-value[b]
No Change	80 (52.6)	99 (67.8)	0.06
Improve	34 (22.4)	25 (17.1)	
Worsen	30 (19.7)	18 (12.3)	
Mixed Change	8 (5.3)	4 (2.7)	

SARS-CoV-2 infection period = defined by the time of EQ-5D-3L survey after symptom onset/positive test
Adults = participants aged ≥16 years
[a] Among participants with EQ-5D-3L surveys at enrollment and SARS-CoV-2 infection period
[b] Boldface indicates significance at the 0.05 level

Supplemental Table 6. Descriptive statistics for health utilities among participants aged ≥8 years with SARS-CoV-2 infection, by infection period, stratified by age and reported presence of symptoms (restricted to 15-30-day and 31-90-day infection periods)

Participants ≥16 years[a]					
SARS-CoV-2 infection period by self-report of symptoms[c]	No.	Mean (SD)	Median (IQR)	Bootstrapped 95% CI	Range
Reported Symptoms					
Overall	341	0.90 (0.15)	1.00 (0.83, 1.00)	0.89,0.92	0.17-1.00
15-30 days (middle)	101	0.87 (0.17)	1.00 (0.81, 1.00)	0.83,0.91	0.17-1.00
31-90 days (late)	240	0.91 (0.13)	1.00 (0.83, 1.00)	0.90,0.93	0.17-1.00
Reported No Symptoms					
Overall	39	0.93 (0.12)	1.00 (0.83, 1.00)	0.89,0.96	0.45-1.00
15-30 days (middle)	16	0.89 (0.15)	0.93 (0.83, 1.00)	0.81,0.95	0.45-1.00
31-90 days (late)	23	0.95 (0.09)	1.00 (0.92, 1.00)	0.91,0.98	0.78-1.00
Participants 8-15 years[b]					
SARS-CoV-2 infection period by self-report of symptoms[c]	No.	Mean (SD)	Median (IQR)	Bootstrapped 95% CI	Range
Reported Symptoms					
Overall	78	0.94 (0.14)	1.00 (0.89, 1.00)	0.90,0.96	0.12-1.00
15-30 days (middle)	21	0.88 (0.23)	1.00 (0.89, 1.00)	0.78,0.96	0.12-1.00
31-90 days (late)	57	0.95 (0.08)	1.00 (0.89, 1.00)	0.93,0.97	0.71-1.00
Reported No Symptoms					
Overall	12	0.94 (0.10)	1.00 (0.84, 1.00)	0.87,0.99	0.71-1.00
15-30 days (middle)	6	0.97 (0.06)	1.00 (1.00, 1.00)	0.92,1.00	0.84-1.00
31-90 days (late)	6	0.90 (0.12)	0.92 (0.83, 1.00)	0.80,0.97	0.71-1.00

SD = standard deviation; IQR = interquartile range; Cl = confidence interval
SARS-CoV-2 infection period = defined by the time of EQ-5D-3L survey after symptom onset/positive test
[a] US value sets for adults (aged ≥16 years) (Shaw JW, Johnson JA, Coons SJ. US valuation of the EQ-5D health states: development and testing of the D1 valuation model. *Med Care*. Mar 2005;43(3):203-20. doi:10.1097/00005650-200503000-00003)
[b] Value sets used to calculate health utilities derived from Spanish children (aged 8-15 years) onset/positive test (Ramos-Goñi JM, Oppe M, Estévez-Carrillo A, et al. Accounting for Unobservable Preference Heterogeneity and Evaluating Alternative Anchoring Approaches to Estimate Country-Specific EQ-5D-Y Value Sets: A Case Study Using Spanish Preference Data. *Value in Health*. 2022;25(5):835-843. doi:10.1016/j.jval.2021.10.013)
[c] Self-reported at least one of the following ≤30 days post-onset/positive test: fever, cough, loss/change in taste/smell, sore throat, muscle/body aches, shortness of breath, diarrhea, fatigue, headache, nasal congestion, vomiting

Supplemental Table 7. Descriptive statistics for health utilities among participants aged ≥8 years with SARS-CoV-2 infection, by infection period, stratified by age, presence of symptoms, and vaccination status prior to infection (restricted to 15-30-day and 31-90-day infection periods)

SARS-CoV-2 infection period by self-report of symptoms[c] and vaccination status[d]	No.	Mean (SD)	Median (IQR)	Bootstrapped 95% CI	Range
**Participants ≥16 years[a]**					
Reported Symptoms—Fully / Partially Vaccinated					
Overall	223	0.91 (0.13)	1.00 (0.83, 1.00)	0.89,0.93	0.31-1.00
15-30 days (middle)	58	0.90 (0.12)	1.00 (0.83, 1.00)	0.87,0.93	0.52-1.00
31-90 days (late)	165	0.91 (0.13)	1.00 (0.83, 1.00)	0.89,0.93	0.31-1.00
Reported No Symptoms—Fully / Partially Vaccinated					
Overall	18	0.95 (0.09)	1.00 (0.88, 1.00)	0.90,0.98	0.78-1.00
15-30 days (middle)	2	1.00 (0.00)	1.00 (1.00, 1.00)	1.00,1.00	1.00-1.00
31-90 days (late)	16	0.94 (0.09)	1.00 (0.84, 1.00)	0.89,0.98	0.78-1.00
Reported Symptoms—Unvaccinated					
Overall	118	0.88 (0.18)	1.00 (0.82, 1.00)	0.85,0.91	0.17-1.00
15-30 days (middle)	43	0.83 (0.21)	0.84 (0.77, 1.00)	0.77,0.89	0.17-1.00
31-90 days (late)	75	0.91 (0.15)	1.00 (0.84, 1.00)	0.88,0.94	0.17-1.00
Reported No Symptoms—Unvaccinated					
Overall	21	0.91 (0.14)	1.00 (0.83, 1.00)	0.84,0.96	0.45-1.00
15-30 days (middle)	14	0.88 (0.15)	0.85 (0.83, 1.00)	0.79,0.94	0.45-1.00
31-90 days (late)	7	0.97 (0.08)	1.00 (1.00, 1.00)	0.91,1.00	0.80-1.00
**Participants 8-15 years[b]**					
SARS-CoV-2 infection period by self-report of symptoms[c] and vaccination status[d]	No.	Mean (SD)	Median (IQR)	Bootstrapped 95% CI	Range
Reported Symptoms — Fully / Partially Vaccinated					
Overall	33	0.95 (0.08)	1.00 (0.89, 1.00)	0.93,0.98	0.71-1.00
15-30 days (middle)	5	1.00 (0.00)	1.00 (1.00, 1.00)	1.00, 1.00	1.00-1.00
31-90 days (late)	28	0.94 (0.08)	1.00 (0.89, 1.00)	0.91,0.97	0.71-1.00
Reported No Symptoms — Fully / Partially Vaccinated					
Overall	6	0.97 (0.06)	1.00 (1.00, 1.00)	0.92, 1.00	0.84-1.00
15-30 days (middle)	2	1.00 (0.00)	1.00 (1.00, 1.00)	1.00, 1.00	1.00-1.00
31-90 days (late)	4	0.96 (0.08)	1.00 (0.96, 1.00)	0.88, 1.00	0.84-1.00
Reported Symptoms — Unvaccinated					
Overall	45	0.92 (0.17)	1.00 (0.89, 1.00)	0.87,0.96	0.12-1.00
15-30 days (middle)	16	0.85 (0.25)	1.00 (0.80, 1.00)	0.72,0.95	0.12-1.00
31-90 days (late)	29	0.96 (0.08)	1.00 (1.00, 1.00)	0.93,0.99	0.72-1.00
Reported No Symptoms — Unvaccinated					
Overall	6	0.90 (0.12)	0.92 (0.83, 1.00)	0.80,0.97	0.71-1.00
15-30 days (middle)	4	0.96 (0.08)	1.00 (0.96, 1.00)	0.88, 1.00	0.84-1.00
31-90 days (late)	2	0.77 (0.08)	0.77 (0.74, 0.80)	0.71,0.83	0.71-0.83

SD = standard deviation; IQR = interquartile range; Cl = confidence interval
SARS-CoV-2 infection period = defined by the time of EQ-5D-3L survey after symptom onset/positive test
[a] US value sets for adults (aged ≥16 years) (Shaw JW, Johnson JA, Coons SJ. US valuation of the EQ-5D health states: development and testing of the D1 valuation model. *Med Care*. Mar 2005;43(3):203-20. doi:10.1097/00005650-200503000-00003)
[b] Value sets used to calculate health utilities derived from Spanish children (aged 8-15 years) onset/positive test (Ramos-Goñi JM, Oppe M, Estévez-Carrillo A, et al. Accounting for Unobservable Preference Heterogeneity and Evaluating Alternative Anchoring Approaches to Estimate Country-Specific EQ-5D-Y Value Sets: A Case Study Using Spanish Preference Data. *Value in Health*. 2022;25(5):835-843. doi:10.1016/j.jval.2021.10.013)
[c] Self-reported at least one of the following ≤30 days post-onset/positive test: fever, cough, loss/change in taste/smell, sore throat, muscle/body aches, shortness of breath, diarrhea, fatigue, headache, nasal congestion, vomiting
[d] Partially vaccinated: received 1 dose of 2 dose SARS-CoV-2 vaccine; Fully vaccinated: received 2 doses of 2 dose SARS-CoV-2 vaccine or 1 dose of 1 dose SARS-CoV-2 vaccine

Supplemental Table 8. Descriptive statistics for health utilities among participants aged ≥8 years with SARS-CoV-2 infection, by infection period, stratified by age and illness severity (restricted to 15-30-day and 31-90-day infection periods)

Participants ≥16 years[a]					
SARS-CoV-2 infection period by illness severity	No.	Mean (SD)	Median (IQR)	Bootstrapped 95% CI	Range
Asymptomatic[c]					
Overall	39	0.93 (0.12)	1.00 (0.83, 1.00)	0.89,0.96	0.45-1.00
15-30 days (middle)	16	0.89 (0.15)	0.93 (0.83, 1.00)	0.82,0.95	0.45,1.00
31-90 days (late)	23	0.95 (0.09)	1.00 (0.92, 1.00)	0.91,0.98	0.78-1.00
Mild[d]					
Overall	312	0.91 (0.14)	1.00 (0.83, 1.00)	0.89,0.92	0.17-1.00
15-30 days (middle)	90	0.89 (0.15)	1.00 (0.81, 1.00)	0.86,0.92	0.26-1.00
31-90 days (late)	222	0.91 (0.14)	1.00 (0.83, 1.00)	0.89,0.93	0.17-1.00
Severe[e]					
Overall	29	0.86 (0.20)	1.00 (0.82, 1.00)	0.79,0.93	0.17-1.00
15-30 days (middle)	11	0.76 (0.28)	0.83 (0.63, 1.00)	0.58,0.9	0.17-1.00
31-90 days (late)	18	0.93 (0.11)	1.00 (0.83, 1.00)	0.88,0.98	0.72-1.00
Hospitalized[f]					
Overall	1	0.84 (NA)	0.84 (0.84, 0.84)	-	0.84-0.84
15-30 days (middle)	0	-	-	-	-
31-90 days (late)	1	0.84 (NA)	0.84 (0.84, 0.84)	-	0.84-0.84
Participants 8-15 years[b]					
SARS-CoV-2 infection period by illness severity	No.	Mean (SD)	Median (IQR)	Bootstrapped 95% CI	Range
Asymptomatic[c]					
Overall	12	0.94 (0.10)	1.00 (0.84, 1.00)	0.88,0.99	0.71-1.00
15-30 days (middle)	6	0.97 (0.06)	1.00 (1.00, 1.00)	0.92,1.00	0.84-1.00
31-90 days (late)	6	0.90 (0.12)	0.92 (0.83, 1.00)	0.8,0.97	0.71-1.00
Mild[d]					
Overall	75	0.94 (0.12)	1.00 (0.89, 1.00)	0.91,0.97	0.12-1.00
15-30 days (middle)	19	0.92 (0.21)	1.00 (0.94, 1.00)	0.8,0.99	0.12-1.00
31-90 days (late)	56	0.95 (0.08)	1.00 (0.89, 1.00)	0.93,0.97	0.71-1.00
Severe[e]					
Overall	3	0.70 (0.26)	0.61 (0.56, 0.81)	0.5,1.00	0.50-1.00
15-30 days (middle)	2	0.56 (0.08)	0.56 (0.53, 0.58)	0.5,0.61	0.50-0.61
31-90 days (late)	1	1.00 (NA)	1.00 (1.00, 1.00)	-	1.00-1.00
Hospitalized[f]					
Overall	0	-	-	-	-
15-30 days (middle)	0	-	-	-	-
31-90 days (late)	0	-	-	-	-

SD = standard deviation; IQR = interquartile range; Cl = confidence interval
SARS-CoV-2 infection period = defined by the time of EQ-5D-3L survey after symptom onset/positive test
[a] US value sets for adults (aged ≥16 years) (Shaw JW, Johnson JA, Coons SJ. US valuation of the EQ-5D health states: development and testing of the D1 valuation model. *Med Care*. Mar 2005;43(3):203-20. doi:10.1097/00005650-200503000-00003)
[b] Value sets used to calculate health utilities derived from Spanish children (aged 8-15 years) onset/positive test (Ramos-Goñi JM, Oppe M, Estévez-Carrillo A, et al. Accounting for Unobservable Preference Heterogeneity and Evaluating Alternative Anchoring Approaches to Estimate Country-Specific EQ-5D-Y Value Sets: A Case Study Using Spanish Preference Data. *Value in Health*. 2022;25(5):835-843. doi:10.1016/j.jval.2021.10.013)
[c] Self-reported none of the following symptoms ≤30 days post-onset/positive test: fever, cough, loss/change in taste/smell, sore throat, muscle/body aches, shortness of breath, diarrhea, fatigue, headache, nasal congestion, vomiting
[d] Self-reported at least one of the following symptoms ≤30 days post-onset/positive test: fever, cough, loss/change in taste/smell, sore throat, muscle/body aches, shortness of breath, diarrhea, fatigue, headache, nasal congestion, vomiting
[e] Self-reported non-hospitalized medical care ≤30 days post-onset/positive test
[f] Self-reported hospitalization ≤30 days post-onset/positive test

Supplemental Table 9. Descriptive statistics for health utilities among participants aged ≥8 years with SARS-CoV-2 infection, by infection period, stratified by age and cohort

Participants ≥16 years[a]					
SARS-CoV-2 infection period by cohort	No.	Mean (SD)	Median (IQR)	Bootstrapped 95% CI	Range
PACC					
Overall	470	0.91 (0.12)	1.00 (0.83, 1.00)	0.90,0.92	0.22-1.00
0-14 days (early)	44	0.89 (0.15)	1.00 (0.83, 1.00)	0.85,0.93	0.22-1.00
15-30 days (middle)	90	0.91 (0.13)	1.00 (0.83, 1.00)	0.88,0.93	0.45-1.00
31-90 days (late)	336	0.91 (0.12)	1.00 (0.83, 1.00)	0.90,0.93	0.31-1.00
C-HEaRT/SEARCh					
Overall	68	0.80 (0.20)	0.83 (0.77, 1.00)	0.76,0.85	0.17-1.00
0-14 days (early)	26	0.81 (0.16)	0.81 (0.77, 0.96)	0.75,0.87	0.44-1.00
15-30 days (middle)	27	0.77 (0.23)	0.81 (0.74, 1.00)	0.68,0.85	0.17-1.00
31-90 days (late)	15	0.86 (0.23)	1.00 (0.83, 1.00)	0.73,0.95	0.17-1.00
Participants 8-15 years[b]					
SARS-CoV-2 infection period by cohort	No.	Mean (SD)	Median (IQR)	Bootstrapped 95% CI	Range
PACC					
Overall	108	0.96 (0.07)	1.00 (0.89, 1.00)	0.94,0.97	0.71-1.00
0-14 days (early)	16	0.95 (0.07)	1.00 (0.89, 1.00)	0.92,0.98	0.83-1.00
15-30 days (middle)	21	0.98 (0.05)	1.00 (1.00, 1.00)	0.95,1.00	0.83-1.00
31-90 days (late)	71	0.95 (0.08)	1.00 (0.89, 1.00)	0.93,0.97	0.71-1.00
C-HEaRT/SEARCh					
Overall	16	0.79 (0.25)	0.81 (0.69, 1.00)	0.66,0.90	0.12-1.00
0-14 days (early)	8	0.88 (0.17)	1.00 (0.72, 1.00)	0.77,0.97	0.61-1.00
15-30 days (middle)	6	0.64 (0.31)	0.67 (0.53, 0.85)	0.40,0.85	0.12-1.00
31-90 days (late)	2	0.86 (0.20)	0.86 (0.79, 0.93)	0.71,1.00	0.71-1.00

SD = standard deviation; IQR = interquartile range; Cl = confidence interval

SARS-CoV-2 infection period = defined by the time of EQ-5D-3L survey after symptom onset/positive test

[a] US value sets for adults (aged ≥16 years) (Shaw JW, Johnson JA, Coons SJ. US valuation of the EQ-5D health states: development and testing of the D1 valuation model. *Med Care*. Mar 2005;43(3):203-20. doi:10.1097/00005650-200503000-00003)

[b] Value sets used to calculate health utilities derived from Spanish children (aged 8-15 years) onset/positive test (Ramos-Goñi JM, Oppe M, Estévez-Carrillo A, et al. Accounting for Unobservable Preference Heterogeneity and Evaluating Alternative Anchoring Approaches to Estimate Country-Specific EQ-5D-Y Value Sets: A Case Study Using Spanish Preference Data. *Value in Health*. 2022;25(5):835-843. doi:10.1016/j.jval.2021.10.013)

Supplemental Table 10. Descriptive statistics for health utilities among participants aged ≥8 years with SARS-CoV-2 infection, by infection period, stratified by age and time of survey completion

Participants ≥16 years[a]					
SARS-CoV-2 infection period by time of EQ-5D-3L completion	No.	Mean (SD)	Median (IQR)	Bootstrapped 95% CI	Range
Enrollment[cd]					
Overall	465	0.91 (0.10)	1.00 (0.83, 1.00)	0.90,0.92	0.26-1.00
0-14 days (early)	61	0.91 (0.09)	0.84 (0.83, 1.00)	0.89,0.93	0.75-1.00
15-30 days (middle)	106	0.91 (0.10)	0.93 (0.83, 1.00)	0.89,0.93	0.55-1.00
31-90 days (late)	298	0.91 (0.11)	1.00 (0.83, 1.00)	0.90,0.93	0.26-1.00
End of follow-up[c]					
Overall	465	0.90 (0.12)	1.00 (0.83, 1.00)	0.89,0.91	0.31-1.00
0-14 days (early)	62	0.91 (0.09)	1.00 (0.83, 1.00)	0.89,0.94	0.77-1.00
15-30 days (middle)	99	0.90 (0.11)	1.00 (0.83, 1.00)	0.88,0.93	0.51-1.00
31-90 days (late)	304	0.90 (0.12)	1.00 (0.83, 1.00)	0.89,0.91	0.31-1.00
Participants 8-15 years[b]					
SARS-CoV-2 infection period by time of EQ-5D-3L completion	No.	Mean (SD)	Median (IQR)	Bootstrapped 95% CI	Range
Enrollment[cd]					
Overall	114	0.96 (0.08)	1.00 (0.89, 1.00)	0.94,0.97	0.61-1.00
0-14 days (early)	22	0.96 (0.06)	1.00 (0.91, 1.00)	0.94,0.99	0.83-1.00
15-30 days (middle)	24	0.97 (0.07)	1.00 (0.97, 1.00)	0.94,0.99	0.83-1.00
31-90 days (late)	68	0.95 (0.09)	1.00 (0.89, 1.00)	0.93,0.97	0.61-1.00
End of follow-up[c]					
Overall	103	0.95 (0.09)	1.00 (1.00, 1.00)	0.94,0.97	0.55-1.00
0-14 days (early)	22	0.96 (0.08)	1.00 (1.00, 1.00)	0.93,0.99	0.71-1.00
15-30 days (middle)	21	0.95 (0.10)	1.00 (1.00, 1.00)	0.90,0.99	0.67-1.00
31-90 days (late)	60	0.95 (0.10)	1.00 (1.00, 1.00)	0.93,0.97	0.55-1.00

SD = standard deviation; IQR = interquartile range; Cl = confidence interval
SARS-CoV-2 infection period = defined by the time of EQ-5D-3L survey after symptom onset/positive test
Mean/median number of days between symptom onset/positive test and EQ-5D-3L completion: early: 8.34/8.50; middle: 23.40/23.00; late: 52.45/50.00
[a] US value sets for adults (aged ≥16 years) (Shaw JW, Johnson JA, Coons SJ. US valuation of the EQ-5D health states: development and testing of the D1 valuation model. *Med Care*. Mar 2005;43(3):203-20. doi:10.1097/00005650-200503000-00003)
[b] Value sets used to calculate health utilities derived from Spanish children (aged 8-15 years) onset/positive test (Ramos-Goñi JM, Oppe M, Estévez-Carrillo A, et al. Accounting for Unobservable Preference Heterogeneity and Evaluating Alternative Anchoring Approaches to Estimate Country-Specific EQ-5D-Y Value Sets: A Case Study Using Spanish Preference Data. *Value in Health*. 2022;25(5):835-843. doi:10.1016/j.jval.2021.10.013)
[c] Among participants with EQ-5D-3L completion at enrollment or end of-follow-up
[d] Values represent health utilities prior to symptom onset/positive-test

Supplemental Table 11. Descriptive statistics for visual analog scale scores among participants aged ≥8 years with SARS-CoV-2 infection, by infection period, stratified by age and time of completion

Participants ≥16 years					
SARS-CoV-2 infection period by time of VAS completion	No.	Mean (SD)	Median (IQR)	Bootstrapped 95% CI	Range
Enrollment[ab]					
Overall	465	83.55 (12.57)	85.00 (80.00, 90.00)	82.43,84.62	10-100
0-14 days (early)	61	83.92 (9.62)	85.00 (80.00, 90.00)	81.43,86.26	60-100
15-30 days (middle)	106	84.21 (11.29)	85.00 (80.00, 90.00)	81.72,86.18	31-100
31-90 days (late)	298	83.24 (13.52)	85.00 (80.00, 90.00)	81.68,84.70	10-100
End of follow-up[a]					
Overall	465	82.64 (13.98)	85.00 (76.00, 90.00)	81.32,84.02	1-100
0-14 days (early)	62	81.87 (15.46)	85.00 (75.00, 90.00)	77.93,85.42	11-100
15-30 days (middle)	99	84.87 (10.73)	87.00 (80.00, 90.50)	82.75,86.84	50-100
31-90 days (late)	304	82.07 (14.55)	85.00 (75.00, 90.00)	80.38,83.60	1-100
Participants 8-15 years					
SARS-CoV-2 infection period by time of VAS completion	No.	Mean (SD)	Median (IQR)	Bootstrapped 95% CI	Range
Enrollment[ab]					
Overall	114	94.99 (7.09)	99.00 (90.25, 100.00)	93.68,96.24	62-100
0-14 days (early)	22	93.91 (6.81)	95.00 (90.00, 100.00)	90.91,96.64	75-100
15-30 days (middle)	24	95.88 (5.81)	99.00 (94.75, 100.00)	93.37,98.04	80-100
31-90 days (late)	68	95.03 (7.62)	99.00 (94.75, 100.00)	93.09,96.78	62-100
End of follow-up[a]					
Overall	103	89.79 (13.20)	91.00 (87.50, 100.00)	87.12,92.14	5-100
0-14 days (early)	22	90.36 (10.77)	90.00 (88.00, 100.00)	85.64,94.45	60-100
15-30 days (middle)	21	90.10 (10.02)	91.00 (85.00, 98.00)	85.95,93.95	62-100
31-90 days (late)	60	89.47 (15.02)	91.50 (87.50, 100.00)	85.52,92.80	5-100

VAS = visual analog scale; SD = standard deviation; IQR = interquartile range; Cl = confidence interval
SARS-CoV-2 infection period = defined by the time of EQ-5D-3L survey after symptom onset/positive test
Mean/median number of days between symptom onset/positive test and VAS completion: early: 8.34/8.50; middle: 23.40/23.00; late: 52.45/50.00
[a] Among participants with VAS completion at enrollment or end of-follow-up
[b] Values represent health utilities prior to symptom onset/positive-test

Supplemental Figure 1. Correlation between health utilities and visual analog scale scores among participants with SARS-CoV-2, by infection period

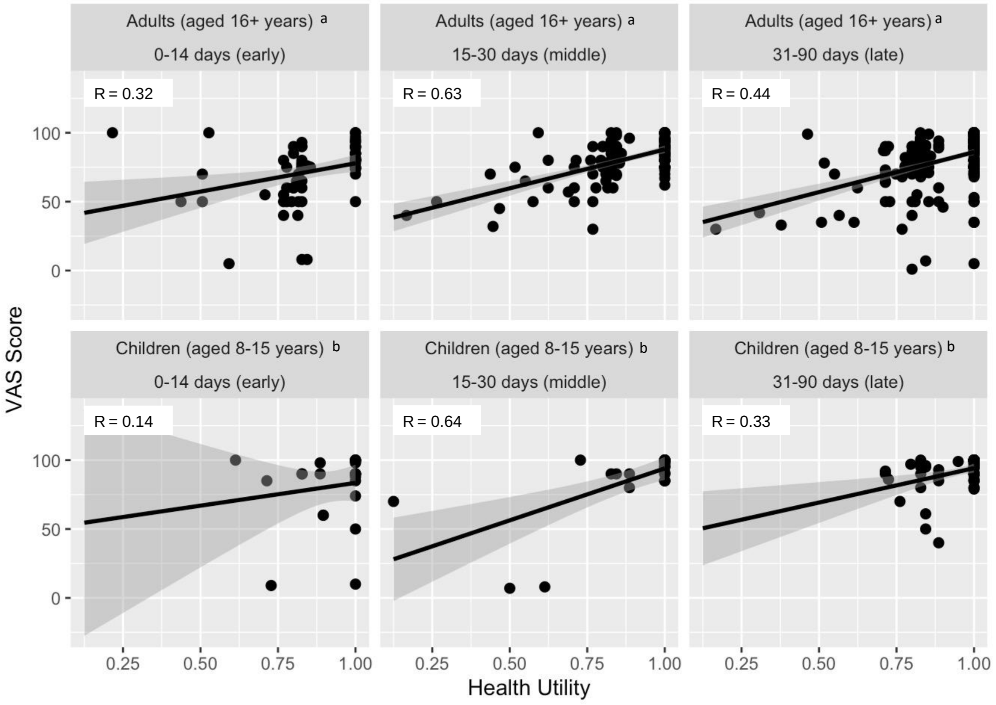

VAS = Visual Analog Scale
SARS-CoV-2 infection period = defined by the time of EQ-5D-3L survey after

[a] US value sets for adults (aged ≥16 years) (Shaw JW, Johnson JA, Coons SJ. US valuation of the EQ-5D health states: development and testing of the D1 valuation model. *Med Care*. Mar 2005;43(3):203-20.doi:10.1097/00005650-200503000-00003)

[b] Value sets used to calculate health utilities derived from Spanish children (aged 8-15 years) onset/positive test (Ramos-Goñi JM, Oppe M, Estévez-Carrillo A, et al. Accounting for Unobservable Preference Heterogeneity and Evaluating Alternative Anchoring Approaches to Estimate Country-Specific EQ-5D-Y Value Sets: A Case Study Using Spanish Preference Data. *Value in Health*. 2022;25(5):835-843.doi:10.1016/j.jval.2021.10.013)
